# Asymmetric voltage amplification using a capacitive load energy management circuit in a triboelectric nanogenerator

**DOI:** 10.1186/s11671-024-03997-8

**Published:** 2024-03-19

**Authors:** Jiwon Jeong, Jiyoung Ko, Jinhee Kim, Jongjin Lee

**Affiliations:** Department of Physics and Research Institute of Natural Science, Gyeongsang National University, Jinju, 52828 South Korea

**Keywords:** Capacitive load, Asymmetric voltage output, Energy management circuit, Triboelectric nanogenerator

## Abstract

We investigated the polarity dependence of a capacitive energy management circuit in a triboelectric nanogenerator (TENG) power system. In a half-wave rectifying circuit, the Simulation Program with Integrated Circuit Emphasis and analytical models show that the charge dump to the load varied depending on the polarity of the rectifying circuit even with the same charge output from TENG. Depending on the polarity of the rectifying circuit, a fast saturation of the direct current (DC) output voltage or a high DC output voltage was obtained. Experiments with a half-wave rectifier and Bennet doubler confirmed our simulation and theoretical results. The charge dump from the minimum capacitance of the separated TENG to the load capacitance and the charge dump from the maximum capacitance of the contacted TENG to the load resulted in asymmetric charging behavior. We concluded that it is necessary to analyze the TENG and the capacitive energy management circuit as a single system rather than considering them as independent units in the rectifying circuit of the TENG. This work can provide insights for the design of triboelectric energy harvesting systems.

**Supplementary Information:**

The online version contains supplementary material available at 10.1186/s11671-024-03997-8.

## Introduction

Triboelectric nanogenerators (TENGs) are extensively researched devices that have shown potential as an alternative power source to batteries and other energy storage devices [1–3]. A TENG generally uses the triboelectric effect to recycle mechanical energy into electrical energy, which is a type of energy harvesting. In the process of friction, the charge can be generated by the process of charge separation that occurs when two different electrical affinity materials come into contact, which can be driven by human movement [4], wind [5], waves [6], or vibrations in the surrounding environment [7]. And in the electric field induction process, charge displacement induced by alternating internal capacitance can generate electrical current [8, 9]. A TENG produces output with high voltages in the hundreds of volts and low currents in the microampere range. TENGs have a structurally high impedance using a dielectric material as the triboelectric layer [10, 11]. This feature helps generate high voltages using frictional charges in the dielectric layer but is inefficient when transferring power to a low-impedance load. An energy management circuit can effectively transfer the generated energy to low-power devices or sensors with low internal impedance. Representative energy management circuits include rectifier circuits [12], oscillator circuits [13], and charge pumps [14]. Improving the energy transfer efficiency in the TENG would allow it to be serve broad applications, and extensive research is underway into such energy management circuits [15–17]. Using energy management circuits in TENGs, such as half-wave rectifiers, full-wave rectifiers, and charge pumps, allows energy to be stored in either charge enhancement mode or voltage enhancement mode [18].

The working mechanism of TENG relies on the triboelectric effect, which occurs when two dissimilar materials come into contact and separate. TENGs are categorized based on their operating principles into four modes: contact-separate mode, slide mode, single electrode mode, and free-standing triboelectric layer mode [19]. All operation modes of TENGs inevitably have a swing between low and high impedance states. After the dielectric layers are charged by triboelectric effects and even after there are no additional charges, structural displacement by mechanical movement renders the capacitance swing that can generate alternating current (AC). To investigate the effect of the intrinsic capacitance change of the TENG on the output while connected to a load through the energy management circuit, we studied a contact-separate TENG (CS-TENG). In this short report, we characterize the asymmetric output of a capacitive load circuit formed by a CS-TENG's intrinsic capacitance through a rectifying circuit. Results demonstrate that a specific connection direction, combined with a rectifying circuit, which enables characteristic charge transfer to a capacitive load depending on the polarity of the rectifying circuit.

## Experimental section

### Numerical procedure

**Fig. 1:**
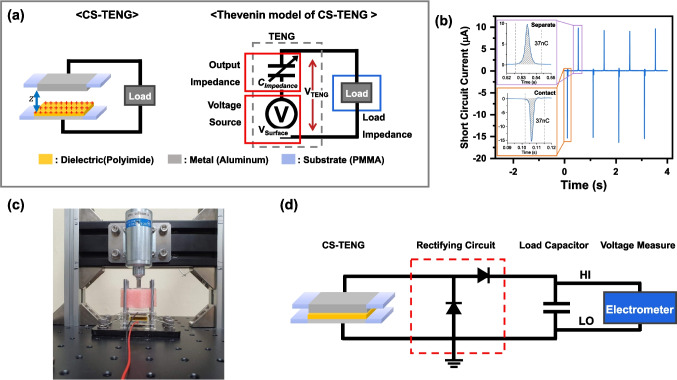
**a** Schematic and Thévenin equivalent circuit model of the CS-TENG. **b** The short circuit current during the contact-separation process. **c** CS-TENG with a solenoid driver for repeated contact-separation process. **d** Experimental schematic of energy management circuit and measurement circuit for output voltage on the load capacitor

The Simulation Program with Integrated Circuit Emphasis (SPICE) calculations for the TENG were used in the Multisim program (National Instruments). Figure 1a shows the lumped-parameter equivalent model of the TENG circuit, which consists of three components: constant surface voltage $\left( {V_{Surface} } \right)$, intrinsic output series impedance $\left( {C_{Impedance} } \right)$, and load impedance. There have been many studies using this model to understand the output of TENG [20–23]. The contact and separation capacitance of the TENG can be calculated from the following equation:

1 $$\begin{array}{*{20}c} {C = \frac{{\epsilon_{0} \times S}}{{\frac{{d_{1} }}{{\epsilon_{1} }} + \frac{{d_{2} }}{{\epsilon_{2} }}}}} \\ \end{array}$$

The parameters utilized in this calculation are listed in Table 1. The contact capacitance of the modeled TENG is 233.7 pF, and the separation capacitance is 0.554 pF. The surface voltage of the TENG was set at 100 V, and the variable capacitor properties were implemented using a voltage-controlled capacitor. The capacitor changed during the transient time of 10 ms of the square wave voltage source. Our SPICE model used an ideal diode model which that has no reverse breakdown voltage.

**Table 1:** Parameters of the TENGs utilized in the SPICE model

Parameter	Value
Permittivity	$\epsilon_{0} = 8.854 \times 10^{12} \;{\text{F}}/{\text{m}}$
Dielectric 1 (PI)	$\epsilon_{1} = 3.3, d_{1} = 50\;\upmu {\text{m}}$
Dielectric 2 (Air)	$\epsilon_{2} = 1.00059, d_{2} = 6.38 \;{\text{mm}}$
Contact Area	$S = 4 \times 10^{ - 4} \;{\text{m}}^{2}$
Surface Voltage	100 V
Contact, Separate transient time	10 ms
$C_{min}$	$0.554\;{\text{pF}}$
$C_{max}$	$233.7\;{\text{pF}}$

### Fabrication and measurement of the model TENG

The experimental TENG created a contact area by attaching a $2 \times 2\;{\text{cm}}^{2}$ polymethyl methacrylate (PMMA) substrate to a $4 \times 4\;{\text{cm}}^{2}$ PMMA substrate. The 50 μm thick aluminum (Al) tape is attached to a $2 \times 2\;{\text{cm}}^{2}$ PMMA substrate as an electrode. Next, 50 μm thick polyimide (PI) tape is adhered to the Al tape electrode to act as the dielectric layer.

### Characterization and measurement

The TENG output characteristics are controlled by a prior charge injection method using an electric field generated by a high-voltage power supply. After exposure to an electric field for a time, a dielectric maintains a specific surface voltage even after removing the field. In our previous experiments, we verified the changes in output and stability depending on the specified electric field [24]. The CS-TENG works by a contact separation process using a solenoid (Shindengen, M080117SS). The electrical output characteristics were measured utilizing an electrometer (Keithley, 6514). For data acquisition, analog output connects to an oscilloscope (Keysight, DSO-2014A). Figure 1b shows the output current of the TENG when only a resistive load is connected. A force sensor (Marveldex, RA18) is affixed to the lower portion of the CS-TENG to detect the application of uniform force (Fig. 1c). The parameters of the CS-TENG measured in the experiment can be found in Table 2. Figure 1c, d show the experimental setup and measurement configuration schematic. The capacitance of the TENG and effective capacitance were measured using an LCR meter (HP4284). The measurement conditions were as follows: frequency: 1 MHz, bias voltage: 0 V, stimulation swing voltage: 0.1 V.

**Table 2:** Output characteristics of experimental CS-TENG and SPICE TENG model

Parameter	CS-TENG
Open circuit voltage ($V_{OC}$)	66.3 V
Short circuit current ($I_{SC}$, pk-pk)	10.1 µA
Amount of charge	16.6 nC
Operating Frequency	1 Hz
Operating force	$0.9\pm 0.05\;{\text{N}}$
Contact Area	2 × 2 cm^2^

The diode in the circuit tested in the model TENG experiments used 1N4007. The breakdown voltage of the diode is 1000 V. The capacitors in the circuit used the following values: half-wave rectifier: $C_{Load} = 47\;{\text{nF}}$, Bennet doubler: $C_{Load} = 47\;{\text{nF}}, C_{Store} = 4.7\;{\text{nF}}$, full-wave rectifier: $C_{Load} = 47\;{\text{nF}}$.

## Results and discussion

In general, the output impedance of the voltage power source has a small value. In contrast, in the case of the TENG, the output impedance is very large, so the measured output voltage of $V_{TENG}$ is heavily influenced by the voltage drop caused by the output impedance. This measured output voltage $V_{TENG}$ is defined as follows:

2 $$\begin{array}{*{20}c} {V_{TENG} = V_{Surface} - V_{Impedance} } \\ \end{array}$$

where $V_{Surface}$ is the surface voltage generated by the charges formed on the surface of the dielectric that constitutes the TENG. $V_{Impedance}$ is the charging voltage of the series capacitance ($C_{Impedance}$) of the TENG, which exhibits a capacitive load characteristic. The current exhibits AC characteristics when only a resistive load is connected, meaning that its direction changes depending on the polarity of the voltage. The amount of charge transferred at each voltage polarity is the same (37 nC), but the current value varies depending on the load resistance (Fig. 1b). In the TENG's $C_{Impedance}$ and $C_{Load}$ connection circuit, when $C_{Impedance}$ changes to $C_{max}$ or $C_{min}$, charge and voltage redistribution occur throughout the circuit, even though the same amount of charge moves at each polarity. This results in an asymmetry in the output voltage of $C_{Load}$ connected under $C_{max}$ or $C_{min}$ condition. When the $C_{Impedance}$ changes, it breaks the voltage equilibrium in the circuit, and charge transfer starts at the electrodes to restore the voltage balance. In our model TENG in Fig. 1a, the capacitance of a TENG is in the range of a few hundred picofarads, and the resistance component in out circuit is only wire and there is negligible resistance. So, the circuit has a small time constant and charges move quickly through the device. After the capacitance becomes stable and the balance of the voltage is established, the charge movement ceases. It is natural to assume that there is no preferred direction in the rectifying circuit to couple the TENG source to the direct current (DC) charge storage capacitor, because the sum of moving charges in each directions are equal as shown in Fig. 1b. However, we propose that there could exist a preferred direction of the charging circuit, and varying $C_{Impedance}$ and $C_{Load}$ should be simultaneously considered.

### Asymmetric output in energy management circuits

To utilize DC output, the TENG output must pass through a rectification circuit. Half-wave rectifiers [25], full-wave rectifiers [26], and Bennet doublers [27] are commonly used as energy management circuits to store the charge of TENGs. The Bennet doubler works as a charge pump and has been found to be effective in improving the output of the TENG [28]. The charge pumping method is well known to increase the voltage using multiple capacitors and switching elements [29].

**Fig. 2:**
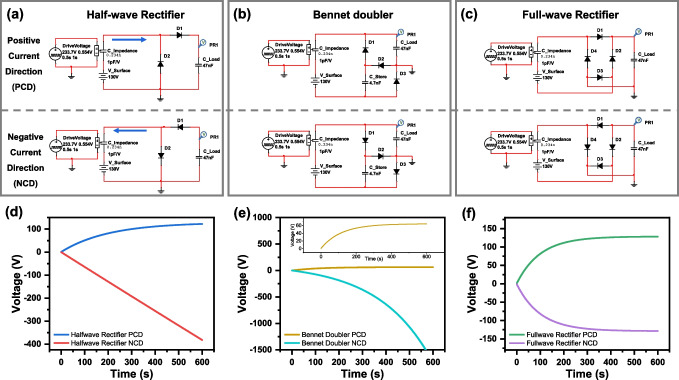
SPICE models of TENG circuits with a capacitive load through **a** half-wave rectifiers, **b** Bennet doublers, and **c** full-wave rectifiers. The voltage appeared across the load capacitor of $C_{Load}$ in the circuit **d** half-wave rectifier, **e** Bennet doubler, and **f** full-wave rectifier

The SPICE is a method for analyzing the electrical characteristics of a circuit by numerical simulation. The use of SPICE allows precise analysis of the TENG. A SPICE model was designed to simulate the change in output impedance to understand how the intrinsic capacitance affects the rectifying TENG circuit. The circuits in Fig. 2a–c show a half-wave rectifier, a Bennet doubler, and a full-wave rectifier, and how to connect the TENG with the output energy-storing load capacitor. We term the ‘positive’ direction as the current flows from the positive voltage of $V_{Surface}$ voltage to $C_{Load}$. The negative current direction means current flows from the negative voltage of $V_{Surface}$ to $C_{Load}$. We investigate the effect of ‘positive’ and ‘negative’ current directions (PCD and NCD) by changing the diode connections. Figure 2d–e show the asymmetric output voltages depending on the polarity of PCD and NCD conditions in each rectifying circuits. The exponential increase in load voltage in the negative current direction of the Bennet doubler is a result of the charge pump effect caused by $C_{Store}$. In the Bennet doubler circuit of Fig. 2e, the additional voltage formed by the charge stored in $C_{Store}$ creates an effect that increases the output voltage of the TENG. This increased output voltage leads to a repeated charge pumping process where more charge is stored in $C_{Load}$ [28, 29]. Symmetrical output appears in the full-wave rectifier shown in Fig. 2f, because it uses both output polarities to charge the load. These results confirm our hypothesis of asymmetric output.

### Analysis of the asymmetric output voltage in the half-wave rectifier

**Fig. 3:**
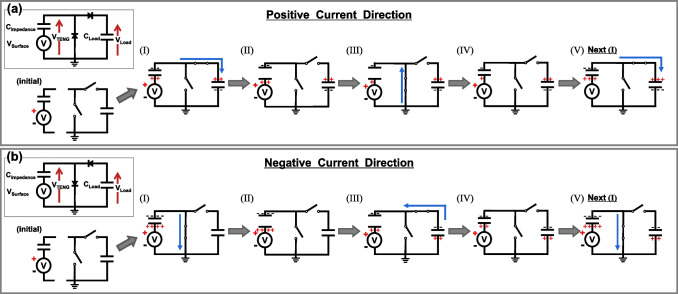
The charging processes of the load capacitor from the TENG connected through the half-wave rectifier in **a** the positive current direction (PCD) and **b** the negative current direction (NCD). TENG separation occurs at step (II) and continues open through step (III), and contact starts at the beginning of step (IV), which applies to **a**, **b**

To analyze the root cause of the asymmetry in the half-wave rectifier, we modeled the TENG and energy management circuits as the TENG’s Thévenin equivalent circuit and ideal switches. Figure 3a, b show our circuit models, which demonstrate charging processes in the load capacitor through the half-wave rectifier diodes that are aligned with or opposed to the positive or negative current directions. We calculate the accumulated charge on the load capacitor as the current repeatedly alternates under the previously defined PCD or NCD condition. The current accumulated on the load capacitor appears as the voltage between the two terminals of the capacitor, which we observe*.*

#### Positive current direction

The charging process in the positive current direction follows the steps shown in Fig. 3a. In the initial state, there are no charges or potential difference in all capacitors, and we assume that all diodes are open. As shown in Fig. 3a-I, when the circuit is connected, the positive value of $V_{TENG}$, derived from Eq. (2), causes the current to flow toward $C_{Load}$. Herein, the amount of charge accumulated on the capacitor is calculated by the composite capacitance of $C_{max}$ and $C_{Load}$ multiplied by $V_{Surface}$ not by $V_{TENG}$:

3 $$\begin{array}{*{20}c} {\Delta Q_{Load} = {\Delta }Q_{Impedance} = \frac{{C_{max} \times C_{Load} }}{{C_{max} + C_{Load} }} \times V_{Surface} } \\ \end{array}$$

In the initial operation, the net charge in the upper floating node is zero. After the current flow stops, all diodes switch off. Separating the TENG reduces $C_{Impedance}$ down to $C_{min}$ by expanding the air gap (Fig. 3a-II). Since $Q_{Impedance}$ remains constant, the reduced capacitance increases $V_{Impedance}$ at a ratio of $C_{max} /C_{min}$. As $V_{Impedance}$ increases, $V_{TENG}$ from Eq. (2) momentarily becomes negative, and current flows from the ground to $C_{Impedance}$ resulting in the discharge of $C_{Impedance}$ (Fig. 3a-III). The remaining charge can be expressed as follows:

4 $$\begin{array}{*{20}c} {Q_{Impedance} = C_{min} \times V_{Surface} } \\ \end{array}$$

In equilibrium, all the diodes become off-state and the following TENG’s contacting process starts. There is an increase in $C_{Impedance}$ and a decrease in $V_{Impedance}$ constrained by a constant charge condition (Fig. 3a-IV). The accumulated net charge in the upper floating nodes is as follows:

5 $$\begin{array}{*{20}c} {Q_{net} = Q_{Impedance} + Q_{Load} > 0} \\ \end{array}$$

The remaining charges in each capacitor provide initial voltage conditions in the repeating step. In the repeating TENG operation stage of Fig. 3a-V, the remaining charges in Eq. (5) are redistributed, and $V_{Impedance}$ and $V_{Load}$ appear. From this stage, $V_{Surface}$ charging Eq. (3) should be modified as follows:

6 $$\begin{array}{*{20}c} {\Delta Q_{Load} = {\Delta }Q_{Impedance} = \frac{{C_{max} \times C_{Load} }}{{C_{max} + C_{Load} }} \times (V_{Surface} - V_{Impedance} - V_{Load} )} \\ \end{array}$$

The term ${\Delta }Q_{Load}$ provides additional charges to $C_{Impedance}$ and $C_{Load}$ resulting in an increase in $V_{Load}$. During the cycling process, the accumulated charges ${\Delta }Q_{Load}$ in Eq. (6) exponentially decreased to zero. Thus, $V_{Load}$ asymptotically approaches $V_{Surface} - V_{Impedance}$.

#### Negative current direction

As shown in Fig. 3b, in the case of the negative current direction, when the TENG is connected, the initial $C_{Impedance}$ is charged by $V_{Surface}$ (Fig. 3b-I). The amount of charge stored in the capacitor ($C_{Impedance}$) is as follows.:

7 $$\begin{array}{*{20}c} {Q_{Impedance} = C_{max } \times V_{Surface} } \\ \end{array}$$

After the $C_{Impedance}$ charging current stops and all diodes are in the off state, the separation process of the TENG decreases $C_{Impedance}$ down to $C_{min}$, which increases $V_{Impedance}$ up to $V_{Surface} \times C_{max} /C_{min}$ (Fig. 3b-II). As $V_{Impedance}$ increases, $V_{TENG}$ reaches a negative voltage and the upper diode turns on, which establishes a composite capacitance charging circuit (Fig. 3b-III). The resulting charge transfer equation is as follows:

8 $$\begin{array}{*{20}c} {\Delta }{Q_{Load} = {\Delta }Q_{Impedance} = \frac{{C_{min} \times C_{Load} }}{{C_{min} + C_{Load} }} \times (V_{Surface} - V_{Impedance} )} \\ \end{array} { }$$

The accumulated charge in the first cycle is just determined by $V_{TENG}$ as expressed in Eq. (2). In this process, the charging current flows in the reverse direction compared with the positive case. When $C_{Impedance}$ and $C_{Load}$ equilibrate, the charging current ceases to flow and the diodes turn off. After turning off all the diodes, the TENG comes into contact as shown in Fig. 3b-IV. The increased $C_{Impedance}$ makes $V_{Impedance}$ to decrease, resulting in $V_{TENG}$ being positive. The total net charge accumulated in the upper floating nodes is as follows:

9 $$\begin{array}{*{20}c} {Q_{net} = Q_{Impedance} + Q_{Load} < 0} \\ \end{array} { }$$

After one cycle of the contact-separation process, the new contact-separation process starts at step V. At that time, there is the stored charge in $C_{Impedance}$ and the corresponding $V_{Impedance}$ Eq. (7) is thus modified as follows:

10 $$\begin{array}{*{20}c} {\Delta Q_{Impedance} = C_{max } \times \left( {V_{Surface} - V_{Impedance} } \right)} \\ \end{array}$$

Note that the increased charge in Eq. (10) should be added to the remaining charge at $C_{Impedance}$, but this total charge should be equal to Eq. (7). Likewise, the $V_{Load}$ charging step of the second cycle and thereafter should be modified from Eq. (8). Considering $V_{Impedance}$ and $V_{Load}$ the charging equation can be expressed as follows:

11 $$\begin{array}{*{20}c} {\Delta Q_{Load} = {\Delta }Q_{Impedance} = \frac{{C_{min} \times C_{Load} }}{{C_{min} + C_{Load} }} \times (V_{Surface} - V_{Impedance} - V_{Load} )} \\ \end{array} { }$$

We note that the difference in Eq. (6) and (11) is $C_{max}$ and $C_{min}$. $V_{Load}$ also increases asymptotically up to $V_{Surface} - V_{Impedance}$ as in the case of positive current direction. However, the charging slope in this negative current direction is low. The charging time constant is $C_{min} /C_{Load}$ whereas that of the positive current direction is $C_{max} /C_{Load}$.

#### Final expression for load voltage

Equation (6) and (11) can be converted to differential equation. And its detailed derivation is shown in Supplementary Information. Two equations can be expressed as a single final form using $V \equiv V_{Load} - V_{TENG}$, as follows:

12 $$\begin{array}{*{20}c} {V_{Load\_f} = V_{TENG} + \left( {V_{Load\_i} - V_{TENG} } \right)e^{ - \alpha t} } \\ \end{array}$$

Because $V_{Load\_i}$ is zero, the following expression is derived:

In the PCD, the equation is expressed as follows:

13 $$\begin{array}{*{20}c} {V_{Load\_f} = V_{TENG} \left( {1 - e^{{ - \frac{{C_{max} }}{{C_{Load} }}t}} } \right) = \left[ {V_{Surface} - V_{Impedance} \left( {C_{min} } \right)} \right]\left( {1 - e^{{ - \frac{{C_{max} }}{{C_{Load} }}t}} } \right)} \\ \end{array}$$

In the NCD, the equation is expressed as follows:

14 $$\begin{array}{*{20}c} {V_{Load\_f} = V_{TENG} \left( {1 - e^{{ - \frac{{C_{min} }}{{C_{Load} }}t}} } \right) = \left[ {V_{Surface} - V_{Impedance} \left( {C_{max} } \right)} \right]\left( {1 - e^{{ - \frac{{C_{min} }}{{C_{Load} }}t}} } \right)} \\ \end{array}$$

The equations provide the final voltage across the load as a function of time, indicating an exponential approach to a steady state determined by the TENG voltage and ratio between $C_{Impedance}$ and $C_{Load}$.

Both charging processes of $V_{Load}$ asymptotically approach $V_{TENG}$ such that $V_{Surface} - V_{Impedance}$. It is important to note that the value of $V_{Impedance}$ is the voltage when the load is connected to the TENG. Thus, the saturation voltage in both cases can be expressed as follows:

15 $$\begin{array}{*{20}c} {V_{Saturation\_PCD} = V_{Surface} - \frac{{C_{min} \times V_{Surface} }}{{C_{max} }} \cong V_{Surface} \quad \left( {t = \infty } \right)} \\ \end{array}$$

16 $$\begin{array}{*{20}c} {V_{Saturation\_NCD} = V_{Surface} - \frac{{C_{max} \times V_{Surface} }}{{C_{min} }} \cong - V_{Surface} \times \frac{{C_{max} }}{{C_{min} }}\quad \left( {t = \infty } \right)} \\ \end{array}$$

The negative current direction has a voltage gain of $C_{max} /C_{min}$, which is typically hundreds or thousands of times.

At the early stage of the charging process PCD and the NCD can be express:

17 $$\begin{array}{*{20}c} {V_{PCD} = V_{Surface} \times \frac{{C_{max} }}{{C_{Load} }} - C_{min} \times \frac{{V_{Surface} }}{{C_{max} }} \times \frac{{C_{max} }}{{C_{Load} }} \cong V_{Surface} \times \frac{{C_{max} }}{{C_{Load} }}\quad \left( {t \ll 1} \right)} \\ \end{array}$$

18 $$\begin{array}{*{20}c} {V_{NCD} = V_{Surface} \times \frac{{C_{min} }}{{C_{Load} }} - C_{max} \times \frac{{V_{Surface} }}{{C_{min} }} \times \frac{{C_{min} }}{{C_{Load} }} \cong - V_{Surface} \times \frac{{C_{max} }}{{C_{Load} }}\quad \left( {t \ll 1} \right)} \\ \end{array}$$

It is worth noting that in the initial stage, the charging amount at $C_{Load}$ is the same in each cycle with different polarity. But as the cycle progressed, the charging amount of NCD is almost constant. Whereas the charging amount in PCD is heavily deteriorated by charged $C_{Load}$.

### Experimental verification in the model TENG

**Fig. 4:**
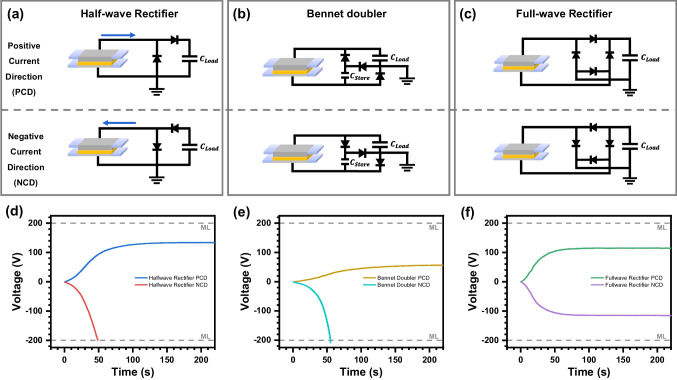
Experimental TENG circuit diagrams with a capacitive load through **a** half-wave rectifiers, **b** Bennet doublers, and **c** full-wave rectifiers. The voltage appeared across the load capacitor of $C_{Load}$ in the circuit **d** half-wave rectifier, **e** Bennet doubler, and **f** full-wave rectifier. **a** and **c**: $C_{Load} = 47\;{\text{nF}}$, **b**:$C_{Load} = 47\;{\text{nF}}, C_{Store} = 4.7\;{\text{nF}}$. *Note*: ML means measure limit

Figure 4a–c confirm our simulation results through model TENG experiments. The experiment used the prior charge injection method to induce a positive polarity surface charge on polyimide [24]. As shown in Fig. 4d–f, the measured voltages ($V_{Load}$) show trends consistent with the corresponding simulation curves. Connecting a half-wave rectifier and a Bennet doubler circuit shows that a specific polarity effectively produces high voltage. Conversely, a full-wave rectifier generates a symmetrical output independent of the polarity as it utilizes both output polarities (Fig. 4f). A half-wave rectifier charges the capacitor $C_{Impedance}$ during one half-period and then transfers the charge to the capacitor $C_{Load}$ during the next half-period. In contrast, a Bennet doubler charges the external capacitor $C_{Store}$ during one half-period and then transfers the charge to the capacitor $C_{Load}$ during the next half-period. A full-wave rectifier utilizes both half-cycles of the input waveform, resulting in same magnitude with different polarity output voltage regardless of the circuit's direction.

**Fig. 5:**
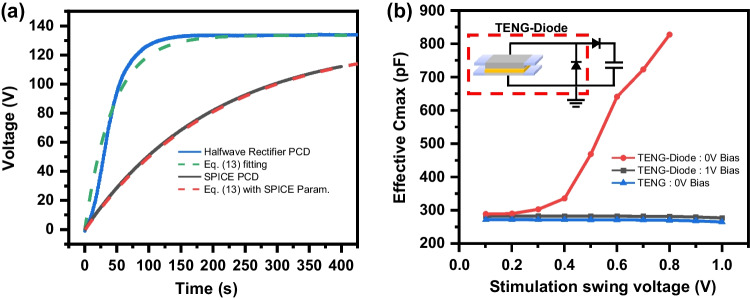
**a** Experimental (blue line), SPICE (black line), and Eq. (13) with measured parameters (red dash line) and experimental fitting with Eq. (13) (green dash line) for the half-wave rectifying circuit in PCD case. **b** The effective $C_{max}$ of the TENG only and TENG with diode circuit (inset) depending on AC stimulation swing voltage

In Fig. 5, charging voltage curves of the half-wave rectifier under the PCD condition were plotted as experimental, SPICE and Eq. (13). Despite using the experimental parameters of the TENG, SPICE and Eq. (13) show very slow saturation characteristics. To verify the cause of slow saturation, the experimental saturation curve was fitted using Eq. (13) and parameters were shown in Table 3. The $C_{max}$ value should be much larger than the measured $C_{max}$ value of the experimental TENG. Since the charging process involves transferring the stored charge from $C_{max}$ to $C_{Load}$, a larger $C_{max}$ value leads to faster saturation. To investigate the origin of the increased $C_{max}$, the capacitance of the TENG and the parallel capacitance of the TENG with the diode in the inset of Fig. 5b were measured as Fig. 5b. The results can be explained that the diode forward junction capacitance and the $C_{Impedance}$ of TENG form the parallel capacitor. This increased effective $C_{max}$ additionally store charges working as $C_{Store}$ in the Bennet doubler. It is worth noting that even when we measured the same parallel circuit, but with a 1 V DC bias, we did not obtain any increase in effective capacitance, but only a $C_{max}$ of TENG. In this case, there can be no charge extraction from junction capacitance, so there is no increase in parallel capacitance value. In our experiment, the diode undergoes large level voltage swing with different polarity. So, effective charge extraction and storing from diode junction capacitance made $C_{max}$ approaching to the fitting parameter value of 1061 pF.

**Table 3:** Parameters used for experimental and SPICE result analysis and fitting values

Parameter	Experimental of PCD	SPICE and Eq. (13) Param	Equation (13) fitting Param
Saturation Voltage	134 V	130 V	134 V
$C_{Load}$	47 nF	47 nF	47 nF
$C_{max}$	272 pF	233.7 pF	1061 pF
$C_{min}$	1.54 pF*	0.554 pF	–

*Measured with 100 kHz frequency

## Conclusions

The output of a TENG with a capacitive load coupled to a rectifying energy management circuit shows asymmetric output characteristics. In the half-wave rectifying circuit, SPICE and analytical models show that the charge dump to the load varied depending on the polarity of the rectifying circuit even with the same charge output from TENG. In rectifying circuits for DC output voltage, effective energy harvesting strategies should be considered: employing PCDs for fast saturation and NCDs for higher voltage. The main cause of this asymmetry is the asymmetric output impedance of the TENG. We conclude that it is necessary to analyze the TENG and the capacitive energy management circuit as a single system rather than considering them as independent units in the rectifying circuit of the TENG. This work can provide insights for the design of triboelectric energy harvesting systems.

### Supplementary Information

**Additional file 1.**

## Data Availability

Data is provided within the manuscript or supplementary information files.

## Abbreviations

TENG: Triboelectric nanogenerators
AC: Alternating current
CS-TENG: Contact-separate mode TENG
SPICE: Simulation program with integrated circuit emphasis
PMMA: Polymethyl methacrylate
Al: Aluminum
PI: Polyimide
PCD: Positive current direction
NCD: Negative current direction
ML: Measure limit
