# Supplementary material for: Asymmetric voltage amplification using a capacitive load energy management circuit in a triboelectric nanogenerator
Source: Discov Nano. 2024 Mar 19;19(1):52. doi: 10.1186/s11671-024-03997-8 (PMC10951180; doi:10.1186/s11671-024-03997-8)
Supplement: Supplementary file 1 — Additional file 1. [file 11671_2024_3997_MOESM1_ESM.docx]

**Supplementary Information**

**Asymmetric voltage amplification using a capacitive load energy management circuit in a triboelectric nanogenerator**

Jiwon Jeong, Jiyoung Ko, Jinhee Kim, Jongjin Lee^*^

Department of Physics and Research Institute of Natural Science, Gyeongsang National University, Jinju 52828, South Korea

* Author to whom correspondence should be addressed (J. Lee)

Tel.: +82-55-772-1401

E-mail address: [bandy1@gnu.ac.kr](mailto:bandy1@gnu.ac.kr)

**Note S1: The derivation of the saturation voltage in the impedance load.**

In this section, we provide a detailed derivation of the behavior of the load voltage in a TENG system. Our focus is to understand how the voltage across the load capacitor changes over time, considering the influence of the TENG.

1. Initial Equation for Charge:

The charge stored in a load impedance can be expressed in terms of load capacitance${(C}_{Load})$ and load voltage $(\Delta V_{Load})$. This is equal to the amount of charge stored by the TENG's intrinsic capacitor $(C_{Impedance}$:$C_{max}$(PCD) or $C_{min}$(NCD)) and the difference voltage across $V_{TENG}-V_{Load}$. The charge is expressed as follows:

$$\begin{aligned} \Delta Q_{Load}=C_{Load}\times\Delta V_{Load}=C_{max}\left( V_{TENG}-V_{Load} \right)\#\left( S SEQ (S \backslash* ARABIC 1 \right) \end{aligned}$$

In the negative current direction, the charge is expressed as follows:

$$\begin{aligned} \Delta Q_{Load}=C_{Load}\times\Delta V_{Load}=C_{min}\left( V_{TENG}-V_{Load} \right)\#\left( S SEQ (S \backslash* ARABIC 2 \right) \end{aligned}$$

1. Derivation for Load Voltage Change:

Derived from the initial charge equation is as follows:

$$\begin{aligned} C_{Load}\times\frac{\Delta V_{Load}}{\Delta n}=C_{Impedance}\left( V_{TENG}-V_{Load} \right)\#\left( S SEQ (S \backslash* ARABIC 3 \right) \end{aligned}$$

This represents the voltage rate change across the load, indicating that it is proportional to the voltage difference between the TENG and the load. $n$ is the number index of one contact separation cycle, $\Delta n=1$.

Rearranging Equation (S3) gives:

$$\begin{aligned} \frac{\Delta V_{Load}}{\Delta n}=\frac{C_{Impedance}}{C_{Load}}\left( V_{TENG}-V_{Load} \right)\#\left( S SEQ (S \backslash* ARABIC 4 \right) \end{aligned}$$

1. Differential Equation:

By defining $V\equiv V_{Load}-V_{TENG}$, $\Delta n=dt$, transform Equation (S4) into a first-order linear differential equation as follows:

$$\begin{aligned} \frac{dV}{dt}=-\alpha V\#\left( S SEQ (S \backslash* ARABIC 5 \right) \end{aligned}$$

where $\alpha=\frac{C_{Impedance}}{C_{Load}}$, This equation describes how the voltage difference between the TENG and the load changes over time.

1. Solving the Differential Equation:

By integrating Equation (S5), we find $\ln\frac{V_{f}}{V_{i}}=-\alpha t$, which leads to the following expression:

$$\begin{aligned} \frac{V_{f}}{V_{i}}=e^{-\alpha t}\#\left( S SEQ (S \backslash* ARABIC 6 \right) \end{aligned}$$

This equation reveals the exponential nature of the output voltage decay over time.


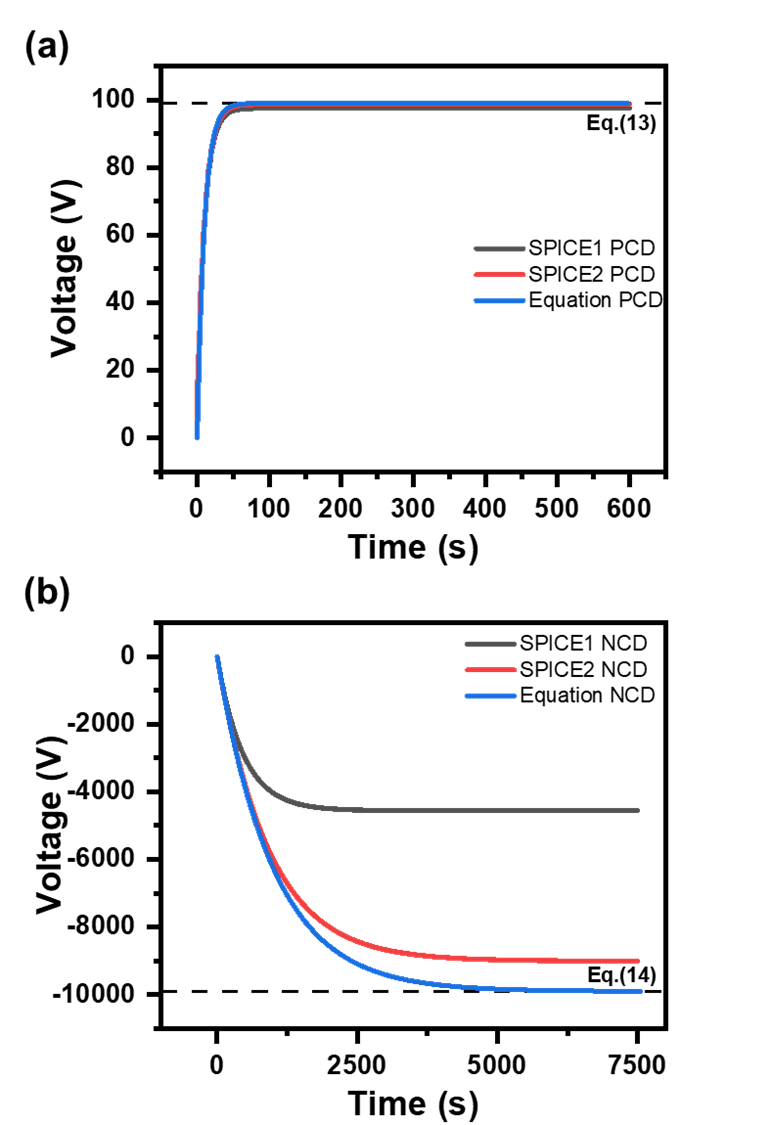


Fig. S1. Comparison of the calculation and SPICE results under (a) the positive current direction and (b) the negative current conditions. The SPICE models are distinguished by the RSHUNT parameter. SPICE1: RSHUNT = 1e12 $\boldsymbol{\Omega}$, SPICE2: RSHUNT = 1e15 $\boldsymbol{\Omega}$.

The SPICE model calculation produces comparable but slightly fewer values from the continuous operation of the diode. This result is related to the RSHUNT parameter in the SPICE program. Inserts a resistance (RSHUNT) from every analog node to ground. This is to eliminate Singular Matrix errors that result from a lack of DC path to ground and, in some cases, to assist convergence [1].

[1] N. Instruments and E. W. Group, NI Multisim User Manual 2009), pp. 1-814
